# HINT1 neuropathy in Lithuania: clinical, genetic, and functional profiling

**DOI:** 10.1186/s13023-022-02541-0

**Published:** 2022-10-14

**Authors:** Matilde Malcorps, Silvia Amor-Barris, Birute Burnyte, Ramune Vilimiene, Camila Armirola-Ricaurte, Kristina Grigalioniene, Alexandra Ekshteyn, Ausra Morkuniene, Arunas Vaitkevicius, Els De Vriendt, Jonathan Baets, Steven S. Scherer, Laima Ambrozaityte, Algirdas Utkus, Albena Jordanova, Kristien Peeters

**Affiliations:** 1 Molecular Neurogenomics Group, VIB Center for Molecular Neurology, VIB, Antwerp, Belgium; 2 Molecular Neurogenomics Group, Department of Biomedical Sciences, University of Antwerp, Antwerp, Belgium; 3 Department of Human and Medical Genetics, Institute of Biomedical Sciences, Faculty of Medicine, Vilnius University, Vilnius, Lithuania; 4 Institute of Clinical Medicine, Faculty of Medicine, Vilnius University, Vilnius, Lithuania; 5 Translational Neurosciences, Faculty of Medicine and Health Sciences, University of Antwerp, Antwerp, Belgium; 6 Laboratory of Neuromuscular Pathology, Institute Born-Bunge, University of Antwerp, Antwerp, Belgium; 7 Neuromuscular Reference Center, Department of Neurology, Antwerp University Hospital, Antwerp, Belgium; 8 Department of Neurology, The Perelman School of Medicine at the University of Pennsylvania, Philadelphia, PA USA; 9 Department of Medical Chemistry and Biochemistry, Medical University - Sofia, Sofia, Bulgaria

**Keywords:** Peripheral neuropathy, Charcot-Marie-Tooth disease, *HINT1*, Neuromyotonia, Lithuania

## Abstract

**Background:**

Recessive loss-of-function variations in *HINT1* cause a peculiar subtype of Charcot-Marie-Tooth disease: neuromyotonia and axonal neuropathy (NMAN; OMIM[#137200]). With 25 causal variants identified worldwide, *HINT1* mutations are among the most common causes of recessive neuropathy. The majority of patients are compound heterozygous or homozygous for a Slavic founder variant (c.110G>C, p.Arg37Pro) that has spread throughout Eurasia and America.

**Results:**

In a cohort of 46 genetically unresolved Lithuanian patients with suspected inherited neuropathy, we identified eight families with *HINT1* biallelic variations. Most patients displayed sensorimotor or motor-predominant axonal polyneuropathy and were homozygous for the p.Arg37Pro variant. However, in three families we identified a novel variant (c.299A>G, p.Glu100Gly). The same variant was also found in an American patient with distal hereditary motor neuropathy in compound heterozygous state (p.Arg37Pro/p.Glu100Gly). Haplotype analysis demonstrated a shared chromosomal region of 1.9 Mb between all p.Glu100Gly carriers, suggesting a founder effect. Functional characterization showed that the p.Glu100Gly variant renders a catalytically active enzyme, yet highly unstable in patient cells, thus supporting a loss-of-function mechanism.

**Conclusion:**

Our findings broaden NMAN’s genetic epidemiology and have implications for the molecular diagnostics of inherited neuropathies in the Baltic region and beyond. Moreover, we provide mechanistic insights allowing patient stratification for future treatment strategies.

## Background

Biallelic loss-of-function alterations in the histidine triad nucleotide-binding protein 1 (HINT1) cause neuromyotonia and axonal neuropathy (NMAN [OMIM#137200]) [1]. Patients with HINT1-deficiency show progressive, predominantly motor polyneuropathy typically starting in the first decade of life, leading to lower limb weakness and gait impairment [2]. Neuromyotonia—peripheral nerve hyperexcitability manifesting as spontaneous muscular activity at rest and delayed muscle relaxation after voluntary contraction—is a clinical hallmark of NMAN. Neuromyotonia is a striking and recognizable feature upon needle electromyography routinely performed in the diagnostic work-up of patients with peripheral neuropathy. So far, the role of HINT1 in the peripheral nervous system is unexplored.

*HINT1* encodes a ubiquitous homodimeric purine phosphoramidase belonging to the evolutionary conserved histidine-triad superfamily. In vitro, HINT1 is a promiscuous enzyme, hydrolyzing diverse AMP-linked substrates [3] and acts as a SUMO1-cleaving Cys-protease [4], yet its endogenous substrate(s) remain unknown. HINT1 has been attributed pleiotropic cellular roles, including regulation of transcription factors involved in tumor progression and apoptosis [5, 6], modulating G-protein coupled receptor signaling [7], and controlling calcium signaling via the store-operated calcium entry pathway [8].

Currently, 25 causal variants have been identified in over 100 NMAN-patients from Europe, Asia and America [1, 2, 9–12]. Haplotype analysis demonstrated founder effects for four of the recurrent *HINT1* mutations in Europe [1, 2, 13] (p.Arg37Pro, p.Cys84Arg, p.Arg95Gln, p.His112Asn) and one in China [9] (p.Cys38Arg) explaining the elevated prevalence of NMAN in certain geographical areas. The ancient Slavic founder allele p.Arg37Pro, present in the majority of NMAN-patients, has a particularly high carrier frequency (1:67-250) in Central and South-East Europe, Russia and Turkey [1, 10, 14]. As a consequence, NMAN ranks among the most frequent forms of axonal neuropathy in those regions.

Most causal NMAN-variants are recurrent missense changes, targeting conserved but also less conserved amino acid residues all over the protein sequence, making it difficult to assess the pathogenicity of a novel variant based on the commonly used criteria like frequency, conservation, or position. Moreover, there is increasing evidence that the disease-causing alterations have differential effects on HINT1 protein stability and function. This has important implications for future therapeutic strategies, as the mutational category will determine a patient’s treatment options. Therefore, functional characterization of novel HINT1 variations benefits both diagnostics and patient stratification. NMAN-associated HINT1 alterations cause a loss of (enzymatic) function, because they either lead to unstable protein or transcript, or affect key residues in the catalytic cleft [1]. Genetic complementation testing in a *HNT1*-knockout (KO) growth deficient yeast strain proved that yeast and human HINT1 orthologs are functionally conserved and that the NMAN-variations abolish this function [1]. Notably, this overexpression system can be used to assess the activity of NMAN-proteins that are degraded in endogenous conditions [13]. Here, we performed the first systematic assessment of HINT1 neuropathy in Lithuania and describe a potential new founder event in the Baltic region.

## Results

### Clinical findings in families with the novel p.Glu100Gly variant

We studied nine families carrying *HINT1* mutations (Table 1). In four of them, a novel variant was found to segregate with the peripheral neuropathy in a homozygous or compound heterozygous state. Their clinical findings are presented below.

**Table 1:** Clinical features of patients with HINT1 neuropathy

ID	*HINT1* genotype	Age at exam (y/gender)	Age at onset (y)	Initial symptom	Neurological examination	Additional findings
Lit1	c.110G>C/ **c.299A>G**	45/M	10	Walking difficulties	Distal limb weakness with gait impairment, calf and intrinsic hand and foot muscle wasting, foot drop, absent Achilles tendon reflexes, decreased proprioception	
Lit2.3	**c.299A>G/** **c.299A>G**	44/M	2	Walking difficulties	Distal limb weakness and muscle wasting, foot drop, gait impairment, frequent fall, foot deformities	
Lit2.4	**c.299A>G/** **c.299A>G**	40/F	12	Walking difficulties	Distal limb weakness with gait impairment, calf and intrinsic foot muscle wasting, foot drop, mild action myotonia	
Lit3.3	c.110G>C/ **c.299A>G**	18/F	4	Walking difficulties	Distal limb weakness with gait impairment, calf and intrinsic hand and foot muscle wasting, foot drop, absent tendon reflexes, muscle cramps	
Lit3.4	c.110G>C/ **c.299A>G**	19/F	4	Walking difficulties	Distal limb weakness with gait impairment, calf and intrinsic hand and foot muscle wasting, foot drop, absent tendon reflexes, muscle cramps, neuromyotonic discharges	
Lit4	c.110G>C/ c.110G>C	21/M	2	Delayed motor milestones with tiptoe walking, spasticity, clumsiness	Distal limb weakness with gait impairment, foot drop, absent Achilles tendon reflex, foot and hand deformities, calf and intrinsic hand and foot muscle wasting, dysarthria, rhinophonia	Learning difficulties
Lit5	c.110G>C/ c.110G>C	21/M	7	Walking difficulties	Distal limb weakness with gait impairment, action myotonia, calf and intrinsic hand and foot muscle wasting, rhinophonia, dysphagia	
Lit6	c.110G>C/ c.110G>C	14/M	5	Walking difficulties	Distal limb weakness with gait impairment, foot drop, absent Achilles tendon reflexes, foot deformities, calf and intrinsic hand and foot muscle wasting, decreased proprioception	Learning difficulties (IQ 78)
Lit7	c.110G>C/ c.110G>C	42/F	8	Walking difficulties	Distal limb weakness with gait impairment, calf and intrinsic hand and foot muscle wasting, contractures, foot drop, foot deformities, dysphagia, dysphonia	Psychogenic seizures, mixed personality disorders, suicidal feelings
Lit8	c.110G>C/ c.110G>C	33/M	5	Walking difficulties	Distal limb weakness with gait impairment, calf and intrinsic hand and foot muscle wasting, foot drop, absent tendon reflexes	
USA1	c.110G>C/ **c.299A>G**	58/M	12		Progressive distal leg and hand atrophy and weakness, severe atrophy in the calves and intrinsic hand muscles bilaterally, foot drop, paresthesia, absent tendon reflexes	Anxiety and depression, recurrent right unilateral headache (brain cavernoma)

The newly identified variant is highlighted in bold

y, years; M, male; F, female

The index patient in family Lit1 was a 45-year-old man who developed slowly progressive weakness of his lower limbs, foot drop, difficulty in walking, and frequent falls beginning at age 10 (Table 1). He underwent bilateral surgical correction of foot deformities at age 30, and developed difficulty to straighten fingers and grasp small objects at age 35. The physical examination at age 45 revealed muscle wasting and weakness in distal upper and lower limbs, bilateral pes cavus, foot drop and atrophy of the intrinsic hand muscles and thenar eminence. He did not complain of sensory impairment. His family history was negative for neuromuscular disorders.

**Fig. 1:**
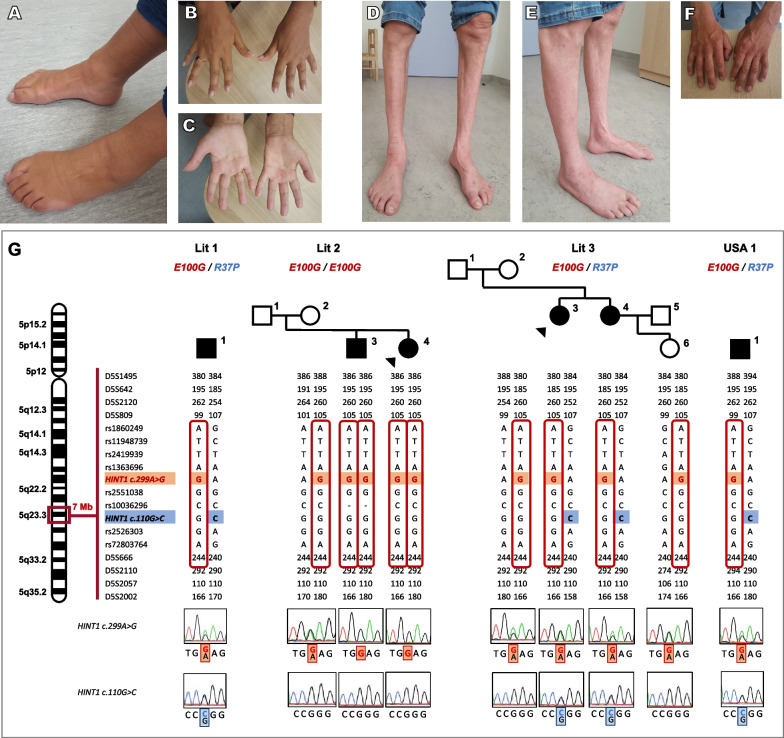
**A**–**F** The clinical phenotype of the patients of family Lit2. At age 40, the index patient had distal atrophy and foot deformities (**A**), atrophy of interdigital muscles (**B**) and atrophy of thenar and hypothenar (**C**). At age 44, her affected brother had calf atrophy and foot deformities shown in frontal and lateral images of the feet (**D**–**E**) and atrophy of interdigital muscles (**F**). **G** Segregation and haplotype analyses of the novel c.299A>G (p.Glu100Gly) *HINT1* variant in three Lithuanian families and one American patient. Sanger sequencing traces show the *HINT1* sequence around the position of each variant in the patients. The minimal shared haplotype on chromosome 5 surrounding the novel variant is indicated in red. The known pathogenic c.110G>C (p.Arg37Pro) founder variant is colored in blue. Squares: males, circles: females, black: affected, white: unaffected. Black triangle indicates the index patient

In family Lit2, a 40-year-old woman presented with gait impairment, distal weakness of lower limbs and bilateral foot drop at the age of 12. Muscle weakness has slowly progressed in a length-dependent manner. Later, she developed difficulties in releasing grip after a strong voluntary hand contraction. She had undergone bilateral achillotomy. The physical examination (Fig. 1A–C) revealed muscle wasting of the lower limbs, and severe distal weakness that was more prominent in the legs. Diminished biceps brachii, triceps brachii, patellar reflexes and absent ankle reflexes were observed. Sensory examination and coordination were normal. Her 44-year-old brother (Fig. 1D–F) was also affected and presented at the early childhood with slowly progressive weakness and muscle wasting of his feet and calves, foot drop, gait impairment and frequent falls. He noticed hand wasting and weakness at the age of 20.

In family Lit3, two affected sisters, now 18 and 19 years old, presented with bilateral foot drop, gait impairment and exercise intolerance at age of 4, followed by gradual progression of muscle weakness and wasting. Patients complained about muscle cramps. Their physical examination revealed muscle wasting of the lower limbs, bilateral foot drop, severe distal weakness that was more prominent in the legs. Diminished reflexes in the upper limbs and absent reflexes in the lower limbs were observed. Sensory evaluation and coordination were normal in both siblings.

Patient USA1 is a 58-year-old man who had a distal hereditary motor neuropathy with a clinical onset at age 12. Regular neurological follow-up since age 36 showed slowly progressive weakness and atrophy predominantly in distal lower limbs, which in the recent years rendered him mostly wheelchair-dependent. The patient now has severe muscle wasting of the lower limbs, bilateral foot drop, absent tendon reflexes. Although initially distal upper limb strength was mildly affected, over the last couple of years, atrophy and weakness in the intrinsic hand muscles increased substantially. Vibration and pinprick sensation had been normal until age 46, but have subsequently declined to the point that vibration is now reduced at the big toes (1 with a Rydell-Seiffer tuning fork) and pinprick is reduced to just below the knees. The patient had prominent muscle twitches and paresthesia in both distal lower and upper limbs; these largely disappeared in his 30s. The patient suffered from chronic anxiety, depression, and insomnia, which was treated with multiple medications including bupropion, buspirone, gabapentin, and melatonin. His two siblings were unaffected.

### Electrophysiological studies

In patient Lit1.1, nerve conduction studies (NCS) performed at age 45 revealed a marked reduction of compound muscle action potentials (CMAPs) in upper limbs and absent in the lower limbs (Table 2). The moderate NCS slowing of the median and the ulnar responses is likely caused by the reduction of CMAPs. Results of the sensory nerve conduction studies were not available. Needle electromyography (EMG) revealed evidence of reinnervation with sparse spontaneous activity in the muscles of the hands.

**Table 2:** Electrophysiological studies of HINT1 patients

ID	Age (y)	Side	Motor								Sensory						EMG	
			Median		Ulnar		Peroneal		Tibial		Median		Ulnar		Sural		Sp. Act.	N. dis.
			CMAP (mV)	CV (m/s)	CMAP (mV)	CV (m/s)	CMAP (mV)	CV (m/s)	CMAP (mV)	CV (m/s)	SNAP (µV)	CV (m/s)	SNAP (µV)	CV (m/s)	SNAP (µV)	CV (m/s)		
Lit1.1	45	L	0.09	18.2	0.39	39.8	ND	ND	ND	ND	NA	NA	NA	NA	NA	NA	+	ND
Lit2.4	40	R	7.6	52.5	6.62	56.4	ND	ND	0.55	35.1	75.0	52.2	57.4	50.5	27.8	45.5	+	+
Lit3.3	18	R	5.15	51.3	7.7	46.8	1.12	34.4	2.9	36.7	92.1	57.5	57.0	61.1	21.5	42.1	+	ND
		L	–	–	–	–	ND	ND	4.35	40.0	–	–	–	–	14.4	43.2		
Lit3.4	19	R	6.4	48.8	3.87	58.8	ND	ND	0.8	34.8	95.0	60.2	78.3	53.9	20.1	36.5	+	+
		L	–	–	–	–	ND	ND	1.77	33.3	–	–	–	–	22.3	35.0		
Lit4	18	R	0.32	47.9	0.78	50.0	ND	ND	0.3	39.7	32.0	53.3	26.0	52.6	–	–	+	+
		L	–	–	–	–	–	–	–	–	–	–	–	–	7.5	42.7		
Lit5	21	R	5.13	49.0	3.2	49.0	0.77	35.9	2.3	36.7	37.3	53.3	14.0	52.9	ND	ND	+	ND
Lit6	15	R	4.3	50.0	1.2	50.0	–	–	–	–	–	–	–	–	–	–	+	+
Lit7	42	R	ND	ND	0.8	50.0	ND	ND	ND	ND	33.3	54.2	31.3	50.0	ND	ND	ND	ND
		L	–	–	–	–	ND	ND	ND	ND	–	–	–	–	ND	ND		
Lit8	33	R	8.7	47.0	4.1	46.2	ND	ND	ND	ND	23.5	47.1	12.8	45.1	ND	ND	ND	ND
		L	7.3	47.8	5.5	44.9	ND	ND	ND	ND	28.3	57.0	10.5	47.4	ND	ND		
USA1	41	R	9.8	52	3.4	57	ND	ND	0.08	40	27.2	58	8.4	63	16.4	34	+	ND
		L	–	–	–	–	–	–	–	–	–	–	–	–	–	–		
USA1	47	R	12.4	50	3.9	57	ND	ND	ND	ND	19.5	61	9.0	50	10.4	41	+	ND
		L	–	–	–	–	–	–	–	–	–	–	–	–	–	–		

Age (y), age at examination in years; R, right; L, left; CMAP, complex motor amplitude potential; CV, conduction velocity; NA, not available; ND, not detected (no response); SNAP, sensory nerve action potential; Sp. Act., spontaneous activity; N. dis., neuromyotonic discharges; –, not measured; +, present

NCS of patient Lit2.4 at age 40 revealed pure motor axonal polyneuropathy in the lower limbs. Sural nerve action potential and conduction velocity were normal. The motor and sensory responses in the upper limbs were normal. EMG showed chronic reinnervation without hallmarks of active denervation. There were several neuromyotonic discharges in the first dorsal interosseous and deltoid muscles. The NCS of her affected brother was not available but was reported as a severe motor axonal neuropathy with absent peroneal CMAPs.

In family Lit3, assessment of peripheral nerves of the hands revealed similar findings in both siblings at the ages of 18 and 19, respectively. CMAPs, sensory nerve action potentials, and conduction velocities were normal. Prolonged distal latencies of the motor median and the motor ulnar nerve on the right were detected. The findings of the NCS of the lower limbs were compatible with axonal motor polyneuropathy, with preserved sensory responses. EMG recording provides evidence of a neurogenic pattern with sparse spontaneous activity. In the younger sister Lit3.4, EMG displayed neuromyotonic discharges in the first dorsal interosseous muscle.

The NCSs of patient USA1 at ages 40 and 47 showed a progressive chronic length-related motor axonal neuropathy. Peroneal motor nerve responses were absent in both studies. The right tibial CMAP amplitude was barely detectable in the first study and undetectable in the follow-up. The right median nerve motor responses were normal, and right ulnar CMAP amplitude was mildly decreased in both studies. Needle EMG showed signs of length-related chronic denervation in the muscles of the right arm that was severe in distal muscles. The sensory nerve responses in the arm and leg were normal at both time points.

### Genetic results

We investigated the occurrence of HINT1 neuropathy in Lithuania by testing a cohort of 46 unrelated index patients with suspected peripheral neuropathy that were excluded for the most common genetic causes: *PMP22* duplication/deletion, *GJB1*, *MFN2* and *MPZ*. Using next-generation sequencing targeting a custom-designed panel of 150 genes associated with Charcot-Marie-Tooth disease and related hereditary neuropathies, we identified biallelic variations in *HINT1* in eight index patients: five affected individuals carried the most common known pathogenic variant NM_005340.7:c.110G>C (p.Arg37Pro) in homozygous state, two patients carried it in compound heterozygous state together with a variant of unknown significance (VUS) NM_005340.7:c.299A>G (p.Glu100Gly), and finally, one patient carried this VUS in homozygous state (Table 1). In addition, in our in-house database of neuropathy patients, we identified an American individual with distal motor neuropathy who carried the same compound heterozygous *HINT1* variation (c.110G>C/c.299A>G). Segregation analysis was performed in two families harboring the unknown c.299A>G variant (Lit2 and Lit3), demonstrating compliance with a recessive inheritance model and uncovering two additional affected individuals (siblings of the index patients) with biallelic *HINT1* variants (Fig. 1G). The three Lithuanian families carrying the novel c.299A>G *HINT1* variation (Lit1-3) originated from different ethnolinguistic regions in the country and were seemingly unrelated. Yet, haplotype analysis of all c.299A>G variant carriers, including the American patient who had a Polish ancestry, demonstrated a shared chromosomal background of 1.9 Mb (D5S809-D5S2110) surrounding the VUS, suggestive of a single mutational origin. We also confirmed that the c.110G>C carriers share the previously established disease haplotype surrounding this known founder allele (Fig. 1G) [1].

In the global population the c.299A>G variation is extremely rare; for instance, in the Genome Aggregation Database (GnomAD v2.1.1) [15] it was observed only once in heterozygous state in a non-Finnish European female (allele frequency 1:251,448). In a cohort of 98 unrelated Lithuanian control individuals, the c.110G>C variation was observed once in heterozygous state (allele frequency 1:196) and the c.229A>G variant was not detected.

The p.Glu100Gly substitution targets a conserved residue (GERP: 3.52) but its predicted effect on HINT1 function is variable (Polyphen-2 v2.2.2r398: probably benign, score = 0.050; SIFT v6.2.1: tolerated, score = 0.38; Mutation Taster: disease causing, probability = 1.00) [16–18].

### Functional characterization

**Fig. 2:**
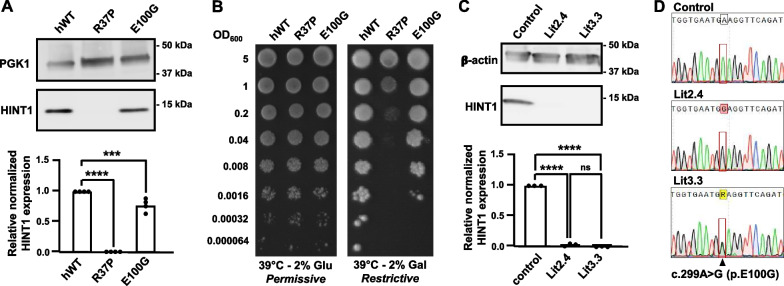
Functional characterization of the identified HINT1 variants. **A** Western blot analysis of protein extract from *HNT1*-deleted yeast strain expressing human HINT1, either wildtype (hWT) or the p.Arg37Pro or p.Glu100Gly alleles. Equal loading was validated with mouse monoclonal anti-PGK1 antibody and relative HINT1 expression was normalized to hWT. The graph represents relative quantification of band intensities of four independent replicates. Note the severe reduction of HINT1 in the p.Arg37Pro-expressing yeast, and the modest reduction of HINT1 in the p.Glu100Gly expressing yeast. **B** Genetic complementation analysis in *HNT1*-deleted yeast strain performed by spot assay. Serial dilutions of the different yeast strains were spotted on minimal media without leucine, supplemented with either 2% glucose or 2% galactose, and incubated at 39 °C for 3 days. Note the reduction of growth of yeast expressing p.Arg37Pro or p.Glu100Gly compared to hWT under the restrictive conditions. **C** Western blot analysis of total protein extracts from HINT1 patient Lit2.4 (p.Glu100Gly/p.Glu100Gly) and patient Lit3.3 (p.Glu100Gly /p.Arg37Pro) cells or control lymphoblasts. Membranes were immunoblotted with polyclonal rabbit anti-human HINT1 antibody. Equal loading was validated with mouse monoclonal anti-β-actin antibody. The bar charts represent the means with standard error of the mean (s.e.m.) of the relative quantification of band intensities of three independent replicates. Note the severe reduction of HINT1 from the patient samples. Statistical one-way ANOVA analysis was performed. ns = not significant; ***p < 0.001; ****p < 0.0001. **D** Sanger sequencing traces of *HINT1* cDNA isolated from peripheral blood mononuclear cells of patient Lit2.4 (p.Glu100Gly/p.Glu100Gly) and patient Lit3.3 (p.Glu100Gly/p.Arg37Pro) or a control individual. The c.299A>G transition is framed. Note the comparable intensity of the peaks at the c.299 position in the compound heterozygous patient

To investigate the impact of the p.Glu100Gly VUS on HINT1 protein function, we performed genetic complementation testing in yeast [1, 13]. Using a vector with a strong, constitutive promotor, we over-expressed human HINT1 transgenes carrying different variants into a *HNT1* KO yeast strain [3]. In contrast to the p.Arg37Pro alteration, which has a detrimental effect on HINT1 protein stability both in yeast and in human cells [1], the p.Glu100Gly variant resulted in substantial HINT1 protein expression in yeast (Fig. 2A). Importantly, this mutated protein appears to retain activity, because it showed (partial) rescue of the restrictive growth deficiency associated with loss of yeast HNT1 (Fig. 2B). Notably, previous study demonstrated that the overexpression system in yeast can lead to enhanced protein expression compared to the endogenous situation [13], therefore increasing artificially the residual activity of the mutant protein, prompting us to interpret the observed rescue effect with caution. Therefore, we analyzed the HINT1 protein levels in peripheral blood mononuclear cells extracted from patients carrying homozygous (Lit2.4) or compound heterozygous (Lit3.3) p.Glu100Gly variants. Immunoblotting analysis showed no detectable HINT1 protein expression in both subjects (Fig. 2C), while the *HINT1* transcript was expressed (Fig. 2D), indicating that the p.Glu100Gly substitution causes severe protein instability in patient cells. The findings were in line with known causal variants (e.g. p.Arg37Pro) where we previously demonstrated that the lack of protein is a result of a post-translational event [1, 13]. In combination with the genetic findings, these functional results provide convincing evidence for pathogenicity of the p.Glu100Gly variant.

## Discussion

This is the first systematic assessment of NMAN in Lithuania, where we identified a total of eight patients from 46 families carrying biallelic missense variations in the *HINT1* gene: the known p.Arg37Pro and/or the novel p.Glu100Gly. The same compound heterozygous variants were identified in a patient from the USA.

In line with previous studies, patients had motor impairment predominating in the distal lower limbs and starting from the first decade of life. Neuromyotonia was reported in some but not all of them. Mild sensory symptoms were only present in a minority of the cases. In addition, some patients displayed atypical features that have been described before, such as developmental delay and intellectual disability [19, 20], speech delay [21], and mood disorder [13, 22]. However, bulbar weakness in the form of dysphagia, rhinophonia, dysphonia, dysarthria has never been reported before.

Haplotype analysis confirmed that the c.299A>G transition in the six identified patients and their relatives originated from a single founder event. The variant is extremely rare, and a search of public genetic variation databases resulted in only a single heterozygous carrier. This individual is (non-Finnish) European, but no further details about ethnicity or nationality are known. As opposed to the most common c.110G>C variant, that was found in 1 out of 98 Lithuanian control individuals, the novel c.299A>G variation was not detected in WGS (n = 50) (unpublished data) and genotype (n = 399) data [23], further exemplifying the rarity of this allele. The American patient carried two recurrent disease-causing *HINT1* variants that cluster in Europe (c.110G>C/c.299A>G) and shared the same disease haplotype as the Lithuanians for both variants. He has Eastern-European heritage, as both his maternal and paternal grandparents emigrated to the US from Poland, a neighboring country of Lithuania and of the Czech Republic, which has among the highest known carrier rates of c.110G>C in Europe (1:182) [14]. Taken together, our results confirm the existence of another pathogenic founder allele in the *HINT1* gene, p.Glu100Gly, that may have originated in the Baltic region. Moreover, our findings expand the geographical distribution of the p.Arg37Pro disease haplotype to the Baltic region [2].

Functional characterization of the novel p.Glu100Gly variant revealed that it causes severe protein degradation in patient cells, providing strong evidence for pathogenicity following a loss-of-function disease mechanism. These results are in line with previous studies showing over 80% of pathogenic HINT1 substitutions to trigger proteasome-mediated protein degradation [1, 13]. Contrastingly, this degradation did not occur to the same extent in the yeast overexpression model, enabling functionality testing of the residual protein. Notably, this experiment proved that the p.Glu100Gly variant gives rise to a HINT1 protein that retains its activity. Therefore, it fits into the same category as other HINT1 variants like p.Cys84Arg, which also renders a protein that remains catalytically active, as seen in an in vitro enzymatic assays, but is degraded in patient cells [1, 24]. This is in contrast to other NMAN-causing mutations that lead to stable but enzymatically dead (e.g. p.His112Asn [1]) or unstable and enzymatically dead (e.g. p.Arg95Gln [13]) protein. To this end, the results of this study have important implications in light of future therapy development. Affected individuals carrying variations like p.Glu100Gly, belong to a subgroup of patients who would benefit from treatment with a pharmacological chaperone that stabilizes the affected yet still catalytically active HINT1 enzyme. Similar approach has been developed for other recessive disorders like cystic fibrosis [25].

On the HINT1 protein structure, p.Glu100 is positioned at the far edge of the dimer interface, a region where multiple NMAN-causing variations cluster (p.Gly93Asp, p.Tyr94Cys, p.Arg95Gln, p.Val97Met). It has been shown before that HINT1 dimerization is crucial to retain enzymatic activity [24]. Through genetic complementation testing in yeast we established that, despite its localization at the dimer interface, the p.Glu100Gly substitution does not seem to abolish the capability of the HINT1 enzyme to form dimers, because the overexpressed protein is functionally active. More likely, the loss of the glutamate side chain at this position could disturb the internal structure of the monomer, similar to other NMAN-variations that preserve dimerization (e.g. p.Cys84Arg, p.Gly89Val), which show reduced thermal stability compared to the wildtype protein [24].

## Conclusions

This study represents the first analysis of HINT1 neuropathy in Lithuania, where we identified a rare novel pathogenic allele (p.Glu100Gly). Functional characterization in yeast and patient cells provided mechanistic insights on how the newly reported substitution leads to loss of HINT1 function. The patients displayed typical symptoms associated with HINT1 neuropathy, including motor impairment in distal lower limbs predominant from the first decade of life, but also some atypical features such as developmental delay and mood problems. Our findings expand the genetic epidemiology of *HINT1*-related disorders.

## Methods

### Patients and evaluation

Patients underwent a routine neurological examination. The family history was taken in all cases. Age of onset was determined by asking about the first neuropathy related symptoms. Nerve conduction studies (NCS) were performed using standard techniques. Clinical data and biological samples were collected for all the patients and their relatives whenever possible.

### Sequencing analysis

Genomic DNA of the Lithuanian patients and their relatives was extracted from peripheral leukocytes using standard methods. Next generation sequencing (NGS) was performed using a custom-designed gene panel of 150 genes associated with Charcot-Marie-Tooth disease and related hereditary neuropathies for one affected person from each family. The panel was designed for Ion AmpliSeq™ technology (Ion Torrent, Thermo Fisher Scientific). The DNA libraries were sequenced on an Ion PGM™ Sequencer (Life Technologies). Bioinformatic analysis was performed including alignment of raw sequence reads to a reference human genome and variant calling on the Ion Torrent Suite™ Server. For identification of disease-causing variants, annotation and filtration of identified sequence variants was performed, using ANNOVAR software [26]. Variants with population frequency over 1% in the Single Nucleotide Polymorphism (dbSNP v137), Genomes Aggregation (gnomAD[15]) and 1000 Genomes Project [27] databases were filtered out. Only variants predicted to affect the coding regions (including non-synonymous, predicted missense, nonsense, splice acceptor and donor site, and insertions or deletions) were selected for further analysis. Several in silico prediction programs (PolyPhen-2 [16], MutationTaster [17], SIFT [18]) were used to predict the functional effect as well as the genomic evolutionary rate profiling (GERP) [28] score. Segregation analysis was performed by Sanger sequencing [29].

Genomic DNA was extracted from peripheral blood sample of the US patient using standard procedures. All three exons as well as the 5’ and 3’ UTR of *HINT1* were screened for variants by Sanger Sequencing. PCR products were purified using ExoSAP-IT™ (Thermo Fisher Scientific, Massachusetts, USA) and sequenced in both directions on a 3730xl DNA Analyzer (Applied Biosystems®, Life Technologies). The resulting electropherograms were analyzed with the Seqman™ II- and Editseq™-software (DNASTAR, Inc., Wisconsin, USA).

### Haplotype analysis

Haplotype sharing analysis for both variants was performed using a previously described panel of STR and SNP markers [13]. STR genotyping was done by capillary electrophoresis of fluorescently labeled amplicons containing the marker region (3730xl DNA analyzer, Applied Biosystems, Foster City, CA, USA). SNP genotyping was performed by Sanger sequencing.

### HINT1 expression plasmids

Yeast expression plasmids carrying human HINT1 (pAG415-HINT1-hWT & pAG415-HINT1-Arg37Pro) were generated in a previous study [2]. Mammalian expression plasmid carrying human HINT1 (pCAGGS-HINT1-hWT) was created at the VIB Protein Service Facility (uGent, Ghent, BE). The different HINT1 variants were introduced with site-directed mutagenesis using KAPA HiFi DNA polymerase (Roche Diagnostics, Basel, CH). After overnight DpnI digestion (New England Biolabs, Ipswich, MA) products were transformed into *E. coli* Mach1 chemically competent cells (ThermoFisher Scientific, Waltham, MA, USA) and validation of the correct incorporation of the missense variant was done by Sanger sequencing of the purified plasmid.

### Yeast strain and transformation

*S. cerevisiae* strain BY8-5c (MATα *ura3-52 his3Δ200 trp1Δ901 lys2-801 suc2-Δ9 leu2-3,112 hnt1Δ::URA3*) [3] was provided by Dr. Brenner, University of Iowa, USA. Yeast cells were cultured in rich medium (YPD). Transformation of BY8-5c with the pAG415GPD expression plasmids carrying one of the HINT1 variants or the human wild-type was done with the LiAc/SS carrier DNA/PEG method [30]. Positive clones were selected in minimal medium without Leucine (SD-Leu) supplemented with 2% glucose.

### Spot assay in yeast

Pre-cultures of the different yeast clones were grown overnight in SD-Leu supplemented with glucose. Absorbance was measured and adjusted to an optical density of OD_600nm_ = 5. Serial dilutions of each culture were spotted in 5ul drops on SD-Leu agar plates supplemented either with 2% glucose or 2% galactose. Plates were incubated for three days at 39 °C.

### Cell line establishment and culture

Peripheral blood lymphocytes were isolated using a Ficol Paque gradient and subsequently transformed with Epstein-Barr virus. After a two-hour incubation at 37 °C, cells were centrifuged and re-suspended in RPMI complete medium (Invitrogen, Carlsbad, CA, USA) supplemented with 1% phytohaemagglutinin. Cells were seeded on a 24-well plate and incubated at 37 °C and 5% CO_2_ for three days. After establishment, lymphoblastoid cells were grown in RPMI complete medium containing 15% fetal bovine serum (FBS, Gibco, Waltham, MA, USA), 1% sodium pyruvate, 1% L-Glutamine (Gibco, Waltham, MA, USA) and 1% penicillin/streptomycin (Gibco, Waltham, MA, USA).

### cDNA analysis

Total RNA was isolated from peripheral blood mononuclear cells using the Universal RNA kit (Roboklon GnmG) according to the manufacturer’s instructions and was subsequently treated with DNAse (TURBO DNA‐free kit, Applied Biosystems). cDNA was synthesized by RT‐PCR with random hexamers using the iScript Advanced cDNA Synthesis Kit (Bio-Rad Laboratories). Full length *HINT1* cDNA was amplified by PCR and the amplicons were Sanger sequenced and analyzed as described above. The sequences of the cDNA primers are available upon request.

### Immunoblotting

Human cells were lysed in RIPA lysis buffer (20 mM Tris–HCl pH = 7.4; 150 mM NaCl; 0.1% Nonidet P-40; 0.5% sodium deoxycholate; 0.1% sodium dodecyl sulfate) supplemented with Halt™ Protease Inhibitor Cocktail (ThermoFisher Scientific, Waltham, MA, USA). Protein concentration was determined with the Pierce BCA protein assay kit (ThermoFisher Scientific, Waltham, MA, USA) and adjusted to 20 µg per sample. Lysates were boiled for five minutes in reducing Laemmli sample buffer (Bio-Rad, Hercules, CA, USA) supplemented with 100 mM 1.4-Dithiothreitol (DTT).

Yeast proteins were extracted following a previously published protocol [31]. Briefly, cells were collected before stationary phase (OD_600nm_ = 1) by centrifugation. Then cells were washed first with 2.0 M LiAc and then 0.4 M NaOH for 5 min on ice. Cells were finally boiled for five min in Laemmli sample buffer (Bio-Rad, Hercules, CA, USA) supplemented with 100 mM DTT.

Proteins were separated in 4–15% Mini-PROTEAN® TGX Stain Free™ Protein gels (Bio-Rad, Hercules, CA, USA) and transferred to a nitro-cellulose membrane (Hybond™-P, GE Healthcare, Chicago, IL, USA) using the semi-dry Trans-Blot® Turbo™ Transfer System (Bio-Rad, Hercules, CA, USA). Membranes were blocked for an hour at room temperature with 5% milk powder diluted in PBS supplemented with 0.1% Tween-20 and then incubated with primary antibody overnight at 4ºC and one hour with a secondary horseradish peroxidase-conjugated antibody at room temperature. Blots were developed with Enhanced Chemiluminiscence ECL Plus™ (ThermoFisher Scientific, Waltham, MA, USA) and imaged with ImageQuant™ LAS 4000 (GE Healthcare, Chicago, IL, USA).

The antibodies used in this study were: polyclonal rabbit anti-human HINT1 antibody (Sigma, San Luis, MO, USA), and to demonstrate equal loading: monoclonal mouse anti-PGK1 antibody (ThermoFisher Scientific, Waltham, MA, USA) or monoclonal mouse anti-β-actin antibody (Sigma, San Luis, MO, USA).

## Data Availability

All data generated or analyzed during this study are included in this published article.

## Abbreviations

AMP: Adenosine monophosphate
BCA: Bicinchonic acid
CMAP: Compound muscle action potential
FBS: Fetal bovine serum
GERP: Genomic evolutionary rate profiling
GJB1: Gap function beta-1 protein
gnomAD: Genome Aggregation Database
HINT1: Histidine Triad Nucleotide Binding protein 1
HNT1: Hit family protein 1
KO: Knockout
LiAc: Lithium acetate
MFN2: Mitofusin-2
MPZ: Myelin protein zero
NCS: Nerve conduction studies
NGS: Next-generation Sequencing
NMAN: Neuromyotonia, axonal neuropathy
PBS: Phosphate buffered saline
PEG: Polyethylene glycol
PGK1: Phosphoglycerate kinase 1
PMP22: Peripheral myelin protein 22
SD-Leu: Synthetic defined medium without leucine
SNP: Single nucleotide polymorphism
STR: Single tandem repeat
SUMO1: Small ubiquitin-like modifier 1
VUS: Variant of unknown significance
YPD: Yeast extract peptone dextrose
